# NF-κB信号通路抑制剂与溶瘤麻疹病毒疫苗株协同抗肺癌的作用及机制

**DOI:** 10.3779/j.issn.1009-3419.2021.102.14

**Published:** 2021-04-20

**Authors:** 茂 夏, 刚 孟, 杰 董

**Affiliations:** 210007 南京，南京大学医学院附属鼓楼医院检验科 Department of Laboratory Medicine, The Affiliated Drum Tower Hospital, Medical School of Nanjing University, Nanjing 210007, China

**Keywords:** 溶瘤麻疹病毒疫苗株, 细胞自噬, NF-κB通路, 细胞凋亡, Oncolytic measles virus vaccine strain, Autophagy, NF-κB pathway, Apoptosis

## Abstract

**背景与目的:**

肺癌已成为我国发病率和死亡率居首位的恶性肿瘤。能自我复制、选择性杀伤肿瘤的溶瘤病毒，是治疗恶性肿瘤的有效策略，而其中溶瘤麻疹病毒疫苗株因其良好的溶瘤效果，且对正常细胞无损伤或微损伤，已进入几项临床试验。本研究旨在探讨核转录因子κB（nuclear factor kappa B, NF-κB）信号通路抑制剂与溶瘤麻疹病毒疫苗株协同抗肺癌的作用及机制。

**方法:**

应用Western blot方法检测MV-Edm感染人肺癌细胞A549和H1299并应用细胞自噬相关的siRNA或者应用NF-кB通路抑制剂PS1145后SQSTM1、p-IκBα、IκBα、PARP及BAX的表达水平，运用流式细胞术分析各组细胞凋亡率的变化，同时采用噻唑蓝[3-(4, 5)-dimethylthiahiazo(-z-y1)-3, 5-di-phenytetrazoliumromide, MTT]法检测各组细胞的存活率。

**结果:**

Western blot结果显示MV-Edm感染人肺癌细胞A549和H1299后，自噬引起NF-κB通路的激活，进而抑制细胞凋亡。抑制细胞自噬可抑制NF-κB通路的激活，MV-Edm感染后p-IκBα表达水平随着感染时间有不同程度的升高，IκBα的表达水平则降低，NF-κB通路抑制剂PS1145可促进人肺癌细胞A549和H1299凋亡（*P* < 0.01），并增强其溶瘤效果。

**结论:**

NF-κB信号通路抑制剂PS1145与溶瘤麻疹病毒疫苗株联合可促进人肺癌细胞A549和H1299凋亡，并增强其溶瘤效果。

## Abstract (en)

**Background and objective:**

Lung cancer is one of the leading causes of cancer-related morbidity and mortality. Oncolytic virotherapy is an emerging therapeutic modality that utilizes replication-competent viruses to destroy cancers. As a powerful tool to kill tumor cells with excellent safety profile, attenuated measles virus of the Edmonston strain (MV-Edm) has been widely applied in the development of tumor therapy and preclinical trials. The aim of this study was to investigate the synergistic effect of nuclear factor kappa B (NF-κB) signaling pathway inhibitor and oncolytic measles virus vaccine against lung cancer and the involved mechanisms.

**Methods:**

Using Western blot to detect MV-Edm infection of A549 and H1299 were infected by MV-Edm alone or used the NF-κB pathway inhibitor PS1145/cell autophagy related siRNA, expression level of p-IκBα, IκBα, PARP and BAX were determined by western blot. Using flow cytometry to analysis the rate of apoptosis, and using MTT [3-(4, 5)-dimethylthiahiazo(-z-y1)-3, 5-di-phenytetrazoliumromide] method to detect the cell survival rate.

**Results:**

Inhibition of cell autophagy could obviously inhibit the MV-Edm infection induced the NF-κB pathway activation in A549 and H1299. In MV-Edm infected A549 and H1299, p-IκBα level increased and IκBα level decreased over infection time, compared with control group. Inhibition of the NF-κB pathway by PS1145 could promote the apoptosis of MV-Edm infected A549 and H1299 and amplify the tumor killing effect.

**Conclusion:**

The combination of NF-κB signaling pathway inhibitor pS1145 and oncolytic measles virus vaccine strains can promote the apoptosis of human lung cancer cells A549 and H1299 and enhance their oncolytic effect.

肺癌在我国各类疾病死因中居首位，而非小细胞肺癌（non-small cell lung cancer, NSCLC）是目前发病率和死亡率最高的恶性肿瘤^[1]^。虽然常规治疗手段如手术、放化疗、靶向治疗等不断发展，但对一些耐药性或难治性终末期肺癌仍然束手无策，迫切需要安全有效可行的治疗手段，如基因/生物治疗、免疫治疗等^[2]^。

具有自我复制功能和良好的肿瘤选择性杀伤作用，同时有良好安全性的病毒被称为溶瘤病毒^[3]^。近10年来在临床试验均有广泛深入的研究，目前至少有6种溶瘤病毒进入了I期/II期临床试验，包括腺病毒（Adenovirus）治疗头颈部肿瘤^[4, 5]^；新城疫病毒（Newcastle disease virus）治疗复发性神经胶质瘤^[6, 7]^；麻疹病毒疫苗株（Measles virus vaccine strain）治疗骨髓瘤^[8]^；此外还有单疱疹病毒（Herpers simplex virus）、呼肠孤病毒（Reovirus）及牛痘病毒（Vaccinia）的临床试验等，均显示出在人体治疗过程中良好的安全性及有效性^[9]^。

麻疹病毒减毒疫苗株（MV-Edmonston, MV-Edm）用于健康未感染人群接种逾60年，未出现致病、致死的报道，拥有可靠的安全记录^[10]^。研究^[11, 12]^发现，麻疹病毒疫苗具有肿瘤选择性杀伤作用，对正常细胞无损伤或微损伤。能否有效提高MV-Edm溶瘤效果，取决于明确其溶瘤机制。我们在既往研究中发现并证实，MV-Edm可诱导NSCLC产生细胞自噬来抑制干扰素或调控细胞色素C的释放来抑制细胞的凋亡，从而介导非Caspase依赖的细胞死亡^[13]^。自噬在病毒的感染和复制中扮演着双刃剑的角色，一方面可以通过直接降解病毒的成分蛋白、激活固有免疫或适应性免疫等机制抵抗某些病毒；另一方面，一些病毒能够诱导自噬来抵抗细胞死亡^[14-16]^。既往研究^[17, 18]^表明细胞自噬可以调节核转录因子κB（nuclear factor kappa B, NF-κB）通路，进而影响细胞内蛋白的表达。提示我们MV-Edm诱导细胞自噬是否亦可以调控NF-κB通路，从而对病毒的溶瘤作用产生影响。

本研究应用Western blot方法检测溶瘤麻疹病毒疫苗株单独感染人肺癌细胞A549和H1299或者使用细胞自噬相关的短干扰RNA（short interfering RNA, siRNA）/NF-κB通路抑制剂PS1145后NF-кB通路的p-IκBα及IκBα以及凋亡相关蛋白PARP及BAX的表达水平，同时运用流式细胞术分析各组细胞凋亡率以及采用噻唑蓝[3-(4, 5)-dimethylthiahiazo(-z-y1)-3, 5-di-phenytetrazoliumromide, MTT]法检测各组细胞的存活率的变化。从体外实验证明溶瘤麻疹病毒疫苗株诱导的细胞自噬可以调控NF-κB信号通路的激活，而抑制NF-κB通路的激活可增加MV-Edm感染诱导的人肺癌细胞A549和H1299的凋亡率，增强其溶瘤作用，提供了NF-κB信号通路抑制剂PS1145联合MV-Edm协同抗肺癌的新策略。并揭示了溶瘤麻疹病毒疫苗株通过细胞自噬调控NF-κB信号通路拮抗肺癌细胞凋亡的溶瘤新机制，为溶瘤麻疹病毒疫苗株的临床转化和应用的进一步优化提供了新思路。

## 1 材料与方法

### 1.1 实验材料

靶向ATG7（Invitrogen, HSS116182），BECN1（Invitrogen, HSS112741）及negative control（Invitrogen, 12935400）的siRNA均购自Thermo Fisher Scientific公司。PVDF膜（Roche, 03010040001），WB Immobilon ECL发光液（Millipore, WBKLS0500）。PS1145（CFAD-P6624-5MG）购自Sigma-Aldrich。抗体：抗GAPDH（bioworld，MB001，1:5, 000稀释），抗ATG7（Cell Signaling Technology，#2631，1:1, 000稀释），抗BECN1（Cell Signaling Technology，#3738，1:1, 000稀释），抗IκBα（Cell Signaling Technology，#4814，1:1, 000稀释），抗p-IκBα（Santa Cruz，sc-52943，1:500稀释）和抗SQSTM1/p62（Abcam，ab109012，1:5, 000稀释）。

### 1.2 溶瘤麻疹病毒疫苗株的扩增

将Vero细胞接种于培养瓶中培养，至融合度为50%-60%时吸去上清。PBS充分洗去细胞表层血清，然后去掉PBS。加入Opti-MEM和MV-Edm，并放入37 ℃培养箱培养至合胞体形成率约为70%时移入32 ℃培养箱培养，当合胞体形成率接近100%时，收集培养皿中的所有细胞于EP管中，反复冻融3次后离心，将上清（即MV-Edm）放入-80 ℃冰箱分装保存备用。

### 1.3 细胞培养

人NSCLC株A549（CCL-185）和H1299（CRL-5803）及非洲绿猴肾细胞株Vero（CCL - 81）均购于中国科学院典型培养物保藏委员会细胞库，以上细胞株均通过STR分型与ATCC细胞库标准分型对比确认。上述细胞使用含有5%胎牛血清，2 mmol/L谷氨酰胺，100 U/L青霉素和1 mg/mL链霉素（均购自Invitrogen）的高糖DMEM培养基培养于37 ℃、5%CO_2_的细胞培养箱。

### 1.4 Western blot

配制不同浓度的SDS-PAGE分离胶（8%-12%）和浓缩胶（5%），每个样品的加样量为30 μg，稳压（浓缩胶80 V 30 min，分离胶120 V约80 min）电泳。转膜：将膜和滤纸按负极-海绵-3层滤纸-凝胶-PVDF膜-3层滤纸-海绵-正极的顺序放置，恒流（110 mA）转膜60 min-80 min不等，将凝胶中的蛋白质转印到PVDF膜上。封闭：将转膜后的PVDF膜放入含5%脱脂奶粉的封闭液中室温封闭1 h。孵育一抗：4 ℃冰箱孵育一抗12 h-16 h。孵育二抗：吸去一抗，洗膜5 min×3次，用相应的HRP标记的二抗室温孵育1 h，再次洗膜5 min×3次。曝光：使用化学发光液在免疫印迹曝光系统上进行曝光获取图像，用Image J软件对条带的密度进行分析，计算各条带的平均密度值。

### 1.5 MTT法

将A549/H1299细胞用0.25%胰酶+0.02% EDTA消化，接种于96孔板，置于37 ℃、5%CO_2_培养箱中培养24 h，每孔中加入不同处理，各浓度设5孔，并设调零孔（只有培养液）和对照孔（培养基+药物溶解剂+细胞）。分别培养不同时间：每孔加入灭菌的5 mg/mL MTT（美国Sigma公司）20 μL，继续培养4 h，弃去培养液，加入DMSO液（美国Sigma公司）150 μL/孔，水平振荡10 min使紫蓝色结晶物充分溶解后置于高通量多功能微板测试（492 nm）测定各孔光吸收值（以*A*表示），以空白孔调零，取4孔平均值，计算出细胞抑制率IR=（1-*A*/*A*0）×100%[*A*：各处理浓度组光吸收值；*A*0：未加处理组（对照组）光吸收值]。

### 1.6 细胞凋亡率检测

采用Annexin V/PI双染法，应用流式细胞仪检测（购自美国BD公司）细胞凋亡率的变化。取对数生长期细胞，用0.25%胰酶+0.02% EDTA消化，接种于12孔板，培养至对数生长期。再使用0.25%胰酶（不含EDTA）消化细胞，进行细胞计数，使每个样品约（0.5-1）×10^6^个/mL细胞，加入结合缓冲液500 μL，重悬细胞；室温、避光条件下加入PI染液5 μL，室温避光孵育5 min-15 min，再加入Binding buffer 450 μL，混匀，使抗体与细胞充分结合，加入1 μL Annexin V-PE染液，孵育15 min，1 h内上机检测。

### 1.7 siRNA转染

以6孔板为例，将细胞以2×10^5^个/孔的密度种板，用不含抗生素的完全培养基继续培养至融合度为50%-60%，去除培养基，Opti-MEM洗2次；取100 nmol/L siRNA加入250 μL Opti-MEM混合稀释，同时取3 μL Lipofectamine 2000加入另一支已装有250 μL Opti-MEM的无RNase EP管中，轻柔混匀并室温静置5 min。将Lipofectamine 2000混合液加入含siRNA的EP管中，混匀并室温静置15 min。去除6孔板内的Opti-MEM，并将Lipofectamine 2000-siRNA混合液加入相应的需要转染的孔中。细胞继续放回37 ℃、5%CO_2_的细胞培养箱中培养4 h后加入完全培养基培养。

### 1.8 统计学处理

采用SPSS 17.0统计软件进行数据分析，计量资料以均数±标准差（Mean±SD）表示，采用*t*检验，计数资料比较采用χ^2^检验，*P* < 0.05表示差异有统计学意义。实验均为3次以上重复的结果。

## 2 结果

### 2.1 溶瘤麻疹病毒疫苗株通过诱导细胞自噬调控NF-κB信号通路

我们既往研究证实MV-Edm可诱导细胞自噬抑制细胞凋亡。MV-Edm诱导的细胞自噬抑制细胞凋亡是否与NF-κB信号通路相关呢？为了进一步探究其中的机制，我们将自噬相关基因ATG7及BECN1的两种siRNA以及无特异性沉默功能的阴性对照Control siRNA转染A549和H1299细胞后感染MV-Edm检测SQSTM1蛋白，自噬通路的重要蛋白SQSTM1，作为一种自噬伴侣受体可以与细胞内泛素化的蛋白结合，同时又与自噬体膜蛋白LC3相互作用从而选择性自噬降解细胞内底物。结果提示ATG7及BECN1的两种siRNA确实可以抑制细胞自噬。与阴性对照组相比，MV-Edm感染A549和H1299细胞可诱导细胞自噬，而抑制了自噬相关基因后IκBα的蛋白水平明显升高，不感染MV-Edm时IκBα的蛋白水平无显著变化（图 1），表明MV-Edm诱导的细胞自噬对A549和H1299细胞感染MV-Edm后NF-κB信号通路的调控有至关重要的作用。

**图 1:**
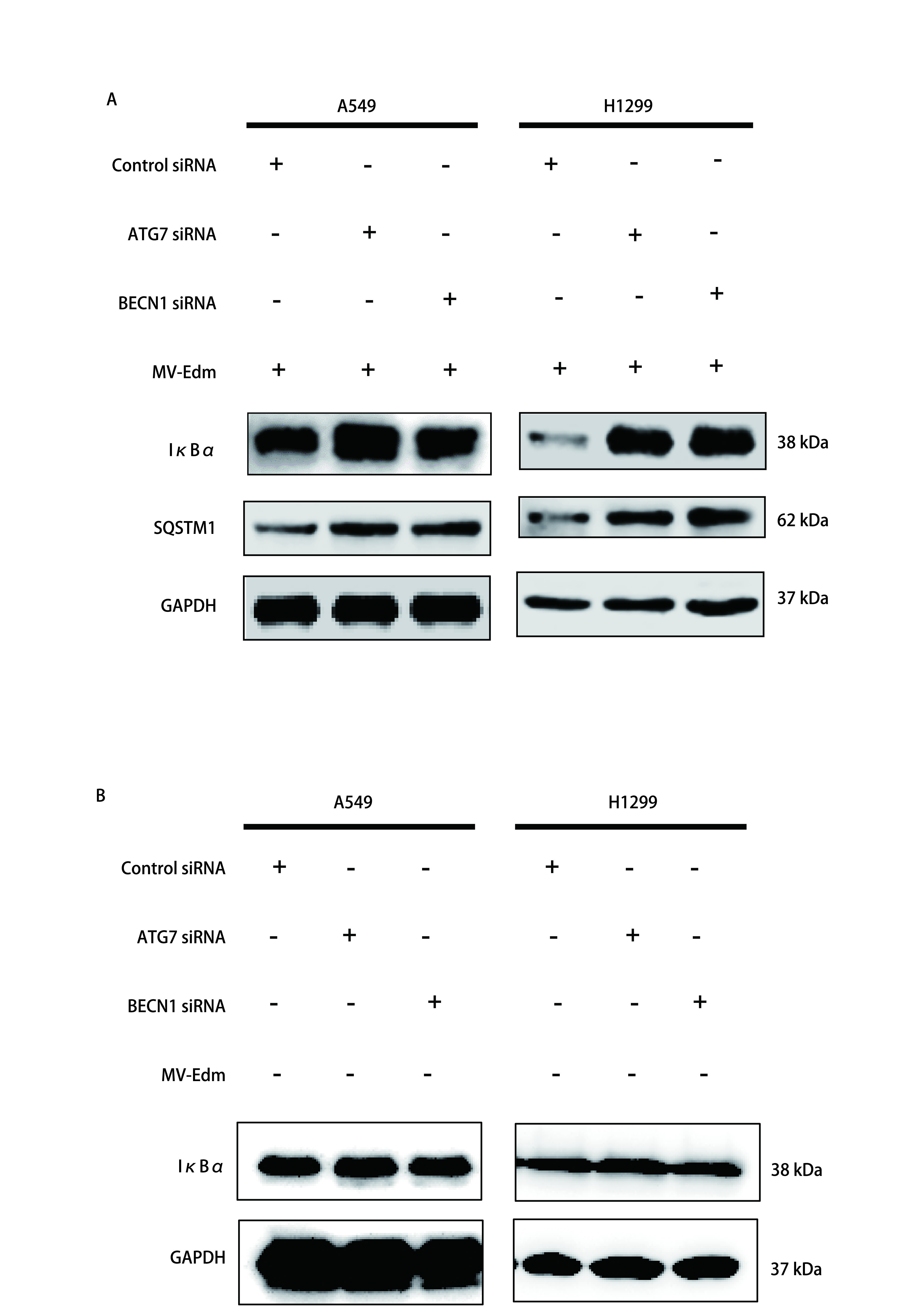
溶瘤麻疹病毒疫苗株通过诱导细胞自噬调控NF-*κ*B信号通路从而抑制细胞凋亡。A：分别转染Control、ATG7、BECN1三种siRNA到A549或者H1299细胞24 h，再用MOI为0.5的MV-Edm感染细胞48 h，去上清，收集细胞提取蛋白，Western blot检测I*κ*B*α*以及SQSTM1的表达，以GAPDH作为内参，以Control siRNA组作为对照；B：分别转染Control、ATG7、BECN1三种siRNA到A549或者H1299细胞，72 h后去上清，收集细胞提取蛋白，Western blot检测I*κ*B*α*以及SQSTM1的表达，以GAPDH作为内参，以Control siRNA组作为对照。 MV-Edm inhibits cell apoptosis by inducing autophagy to regulate NF-*κ*B signaling pathway. A: A549 and H1299 cells transfected with siRNA targeting ATG7, or BECN1 or with nontargeting control siRNA for 24 h were infected with MV-Edm (MOI 0.5) for 48 h, I*κ*B*α*, SQSTM1 and GAPDH was monitored by immunoblotting; B: A549 and H1299 cells transfected with siRNA targeting ATG7, or BECN1 or with nontargeting control siRNA for 72 h, I*κ*B*α*, SQSTM1 and GAPDH was monitored by immunoblotting. NF-*κ*B: nuclear factor kappa B; MOI: multiplicity of infection

### 2.2 MV-Edm感染NSCLC诱导NF-κB信号通路的激活，应用IκB激酶（IκB kinase, IKK）抑制剂PS1145可以抑制NF-κB信号通路的激活

静息状态下，NF-κB二聚体与其抑制蛋白IκB结合以无活性状态贮存于细胞质中。IKK（即Ikk复合体）触发诱导刺激，NF-κB与IκB解离，IκB蛋白即发生磷酸化既成为p-IκB。释放NF-κB二聚体进入细胞核，作用于相应基因的启动子，作为调节炎症反应、氧化应激和免疫的主要核转录因子。我们将MV-Edm感染A549和H1299细胞，分别在不同时间点后收集细胞用Western blot方法检测胞内p-IκBα和IκBα的表达情况既观察NF-κB通路的激活情况。结果如图 2所示，胞内p-IκBα蛋白量随着MV-Edm感染时间的延长逐渐升高，胞内IκBα蛋白量随着MV-Edm感染时间的延长逐渐降低，提示MV-Edm感染A549和H1299细胞后，激活细胞内的NF-κB信号通路。而当我们用IKK抑制剂PS1145抑制NF-κB信号通路后，p-IκBα蛋白量较PS1145未处理组明显降低，而IκBα则明显升高，提示PS1145确实可以显著抑制NF-κB信号通路的激活。上述结果说明MV-Edm感染NSCLC诱导NF-κB信号通路的激活，应用IKK抑制剂PS1145可以抑制NF-κB信号通路的激活。

**图 2:**
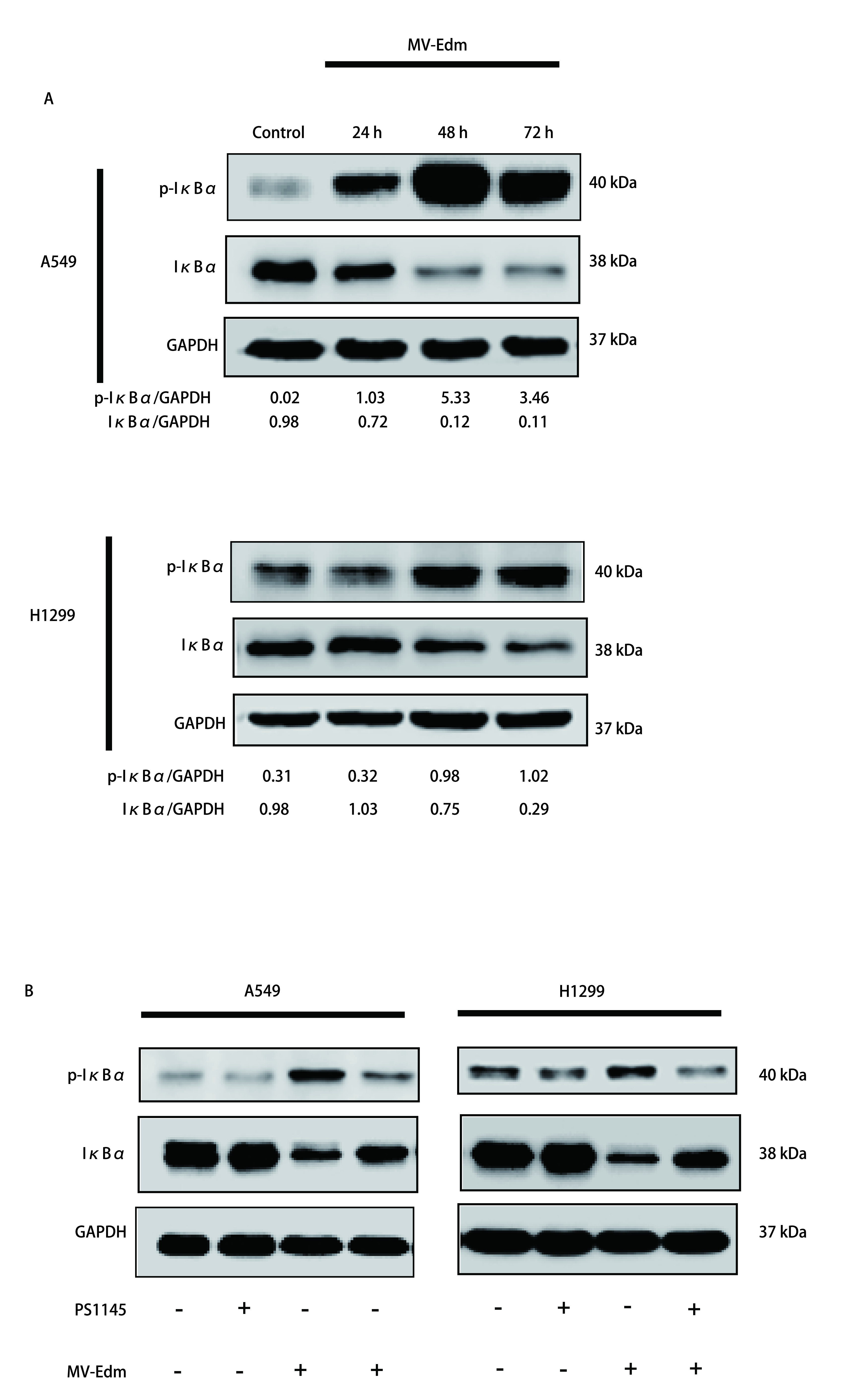
MV-Edm感染肺癌细胞诱导NF-*κ*B信号通路的激活，应用IKK抑制剂PS1145可以抑制NF-*κ*B信号通路的激活。A：用MOI为0.5的MV-Edm分别处理A549和H1299细胞，在24 h、48 h和72 h收集细胞提取蛋白，Western blot检测p-I*κ*B*α*和I*κ*B*α*的表达，以GAPDH作为内参，以不感染病毒作为对照。Image J软件统计灰度比。图中Western blot结果选取自两次独立实验结果之一；B：用NF-*κ*B通路抑制剂PS1145（10 *μ*m/L）分别预处理A549和H1299细胞，3 h后感染MOI为0.5的MV-Edm，48 h后收集细胞提取蛋白，Western blot检测p-I*κ*B*α*和I*κ*B*α*的表达，以GAPDH作为内参，以不感染病毒或/和不做PS1145预处理作为对照。 MV-Edm infection of lung cancer cells induces activation of NF-*κ*B signaling pathway, and application of IKK inhibitor PS1145 can inhibit the activation of NF-*κ*B signaling pathway. A: The level of p-I*κ*B*α* and I*κ*B*α* was monitored by immunoblotting of A549 and H1299 cells after infection by MV-Edm (MOI 0.5) at 24 h, 48 h, and 72 h; B: The level of p-I*κ*B*α* and I*κ*B*α* was monitored by Western blot 48 h after infection of MV-Edm infection with or without IKK inhibitor PS1145.

### 2.3 抑制NF-κB信号通路能促进MV-Edm感染诱导的细胞凋亡

上述研究发现，MV-Edm可能通过诱导细胞自噬调控NF-κB信号通路从而抑制细胞凋亡，那么通过抑制NF-κB信号通路是否可以诱导凋亡促进溶瘤呢？我们通过IKK抑制剂PS1145抑制NF-κB信号通路，进一步检测细胞的凋亡情况，结果如图 3所示，PS1145抑制NF-κB信号通路后感染MV-Edm，A549和H1299细胞的细胞凋亡显著增多（*P* < 0.01）。上述结果说明抑制NF-κB信号通路能促进MV-Edm感染诱导的细胞凋亡。

**图 3:**
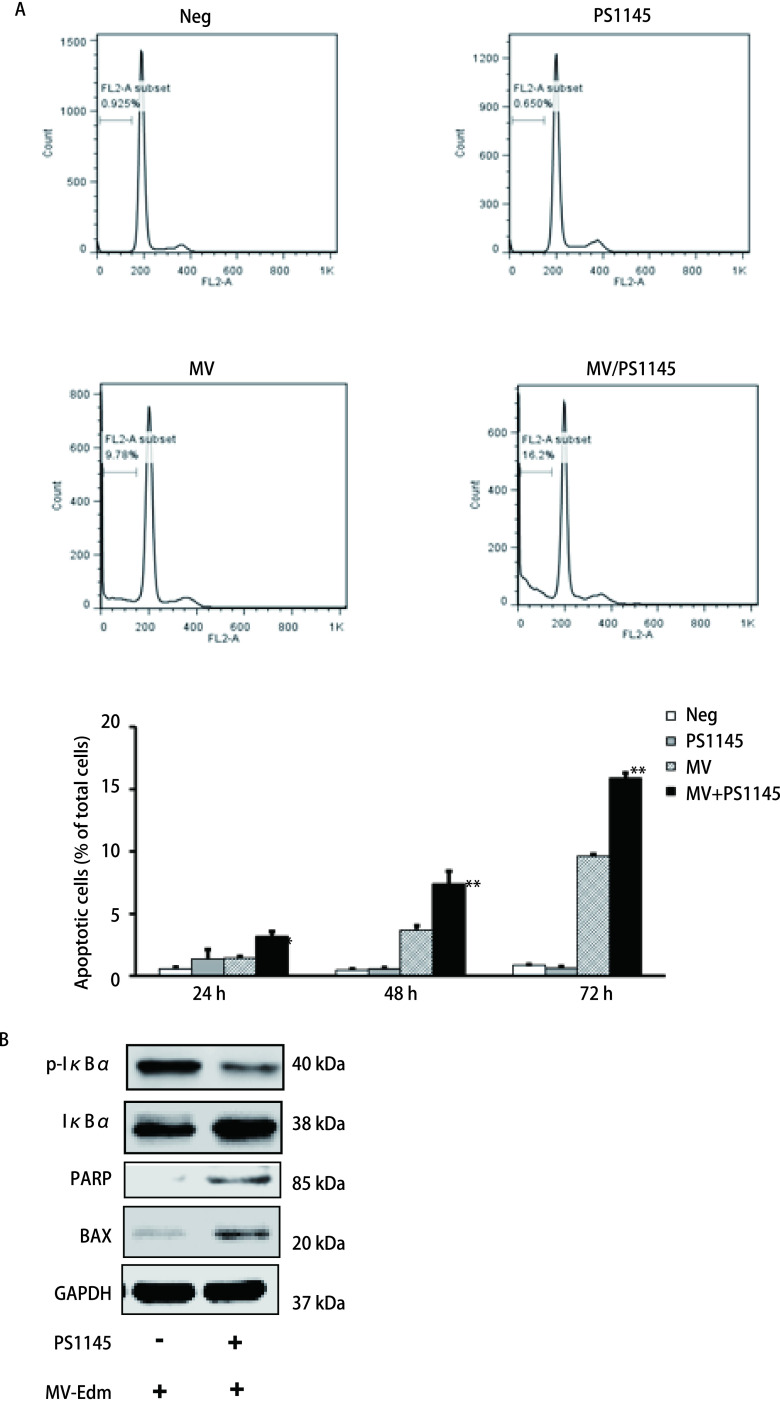
抑制NF-*κ*B信号通路可促进MV-Edm感染诱导的细胞凋亡（*P* < 0.01）。A：用NF-*κ*B通路抑制剂PS1145处理A549细胞，3 h后感染MOI为0.5的MV-Edm，48 h后用Annexin V/PI双染法流式细胞仪检测A549细胞的凋亡情况并做统计分析，以不感染病毒或/和不做PS1145预处理作为对照；B：用NF-*κ*B通路抑制剂PS1145预处理A549细胞，3 h后感染MOI为0.5的MV-Edm，48 h后收集细胞提取蛋白，Western blot检测p-I*κ*B*α*、I*κ*B*α*、PARP以及BAX的表达，以GAPDH作为内参，以不做PS1145预处理作为对照。 Inhibition of NF-*κ*B signaling pathway promots apoptosis induced by MV-Edm infection (*P* < 0.01). A: A549 cells pretreated with PS1145 for 3 h were infected with MV-Edm (MOI 0.5) for at 24 h, 48 h, and 72 h, and cell apoptosis was quantified using Annexin V/PI; B: The level of p-I*κ*B*α*, I*κ*B*α*, PARP and BAX was monitored by immunoblotting 48 h after infection of MV-Edm infection with or without IKK inhibitor PS1145.

### 2.4 抑制NF-κB信号通路可以增强MV-Edm的溶瘤效果

既然抑制NF-κB信号通路可以促进MV-Edm感染细胞的凋亡，因此NF-κB信号通路也可能会影响MV-Edm感染细胞的存活情况。我们通过IKK抑制剂PS1145抑制NF-κB信号通路，感染MV-Edm后不同时间点检测A549和H1299细胞的溶瘤效果，结果如图 4所示，应用PS1145抑制NF-κB信号通路后感染MV-Edm，A549和H1299细胞的存活率明显降低（*P* < 0.01）。上述结果说明NF-κB信号通路抑制剂与溶瘤麻疹病毒疫苗株联用可以增强MV-Edm的溶瘤效果。

**图 4:**
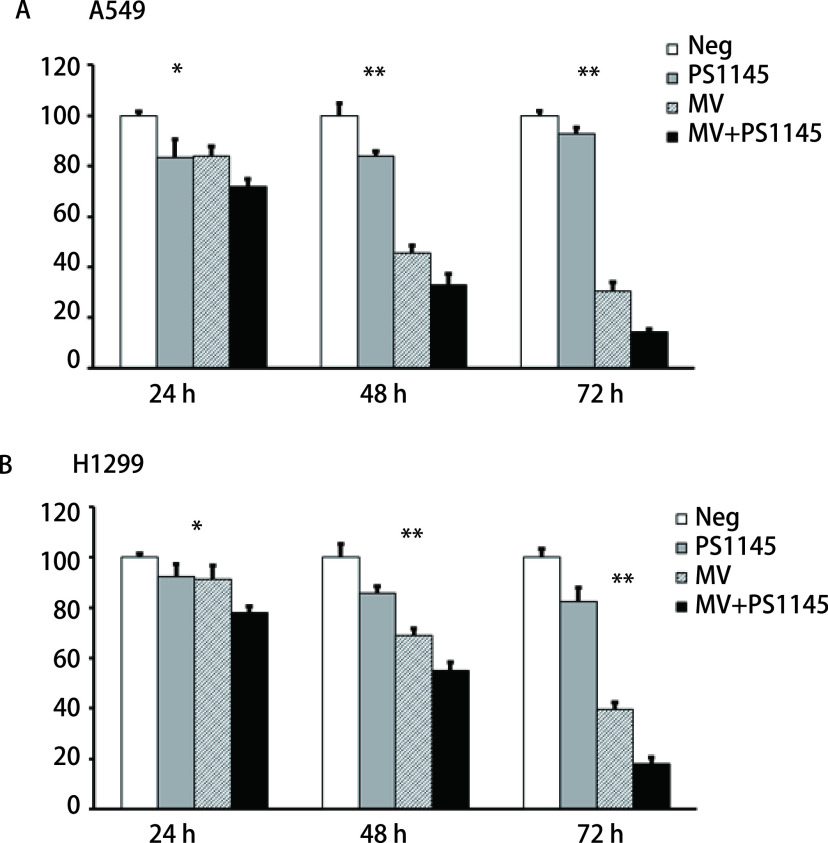
抑制NF-*κ*B信号通路可以增强MV-Edm的溶瘤效果（*P* < 0.01）。用NF-*κ*B通路抑制剂PS1145分别预处理A549和H1299细胞，3 h后感染MOI为0.5的MV-Edm，48 h时后用MTT法检测A549和H1299细胞的存活率，以不感染病毒或/和不做PS1145预处理作为对照。 Inhibition of the NF-*κ*B signaling pathway can enhance the oncolytic effect of MV-Edm(*P* < 0.01), A549 and H1299 cells pretreated with PS1145 for 3 h were infected with MV-Edm (MOI 0.5) for at 24 h, 48 h, and 72 h, and cell death was quantified using MTT. ^*^P < 0.05; ^**^P < 0.01

## 3 讨论

尽管溶瘤麻疹疫苗株MV-Edm因其可靠的安全性和优越的溶瘤效果已进入多项临床试验，但是MV-Edm的具体溶瘤机制至今尚未阐明。而明确其溶瘤机制，对于不断优化溶瘤病毒治疗策略具有极其重要的意义。本研究证实了MV-Edm利用其介导的细胞自噬调控NF-κB信号通路从而拮抗细胞凋亡，并进一步应用NF-κB信号通路抑制剂PS1145与溶瘤麻疹病毒疫苗株联合促进人肺癌细胞A549和H1299凋亡，增强其溶瘤效果。本研究发现的MV-Edm溶瘤新机制，为进一步优化MV-Edm的溶瘤病毒治疗提供了新思路。

细胞凋亡曾被认为是MV-Edm溶瘤作用的主要机制。在MV-Edm治疗人恶性神经胶质瘤和乳腺癌的研究中，感染的肿瘤细胞观察到了凋亡特异性改变^[19, 20]^。但是，凋亡在介导MV-Edm溶瘤中的作用尚没有深入探讨，也不清楚是否存在其他溶瘤方式，而阐明这些机制对于优化MV-Edm的溶瘤策略具有重要意义。实际上，在以往的相关研究中MV-Edm感染肿瘤细胞导致的凋亡都发生在感染晚期。在MV-Edm感染的肿瘤细胞中，细胞凋亡在溶瘤过程中扮演何种角色至今并未阐明。

而NF-κB在癌症中发挥的作用至今仍没有完全阐明。活化的NF-κB在诸如淋巴瘤、肝癌、乳腺癌、结肠癌和胰腺癌等多种癌症研究中被发现^[21]^。NF-κB通路发现与肿瘤的复发、药物抵抗和不良预后相关^[22]^。此外，也有研究发现NF-κB通路的上游激活物或相关转录因子也具有抗肿瘤作用^[23]^。因此，NF-κB究竟是肿瘤抑制还是促进，抑或是与肿瘤类型相关，抑或是抗瘤治疗的潜在干扰因素都需要进一步的研究。在本研究中，我们发现MV-Edm感染肺癌细胞A549和H1299会激活其NF-κB通路从而抵抗细胞凋亡，而抑制NF-κB通路会增强其溶瘤效果。

近年来越来越多的研究表明，细胞自噬与细胞凋亡通路之间存在许多交叉点从而能够相互影响。研究表明，凋亡相关蛋白Bcl-xL或者Bcl-2能与细胞自噬相关蛋白BECN1相结合从而拮抗细胞自噬^[24]^；凋亡关键分子Caspase-3可以通过裂解自噬相关蛋白BECN1从而抑制细胞自噬^[25]^；自噬相关蛋白BECN1通过抑制Caspase-8的降解或凋亡分子Bid的激活从而拮抗凋亡^[26]^；线粒体上的Bcl-xL能够与自噬相关蛋白ATG5的裂解片段相互作用进而控制细胞色素C的释放到胞浆中诱发细胞凋亡等^[27, 28]^。这为本研究进一步探讨MV-Edm感染肿瘤细胞诱导的细胞自噬与细胞凋亡提供了思路和理论基础。

既往许多研究提示细胞自噬很可能通过降解衰老或损伤的细胞器、蛋白质等缓解外界刺激因素对细胞凋亡的诱导从而保护细胞免于或者延缓死亡^[29]^。Joubert小组^[30]^发现基孔肯雅病毒通过诱导自噬延缓Caspase依赖的细胞凋亡；McLean小组发现黄病毒感染上皮细胞会诱导细胞自噬从而保护细胞抵抗凋亡^[15]^。Richetta等^[31]^的研究表明，MV-Edm感染人宫颈癌细胞HeLa所诱导的细胞自噬能抵抗细胞凋亡。我们既往研究也提示MV-Edm通过诱导损伤线粒体自噬控制细胞色素C释放到胞浆中从而拮抗凋亡关键分子Caspase等诱发的细胞凋亡^[13]^。同样的，本研究结果也证实，在细胞自噬被抑制后感染MV-Edm诱导的NF-κB信号通路激活受抑制，而抑制NF-κB信号通路会使细胞凋亡显著增加。这些研究结果也许就能部分解释在以往的研究中为什么细胞在感染MV-Edm的相对晚期才会有出现凋亡特征。MV-Edm诱导的自噬可以拮抗细胞凋亡也许还存在别的可能机制，这需要更进一步的研究。为了提高MV-Edm对不同种肿瘤细胞的溶瘤作用，应用到不同种肿瘤的临床治疗，仍然需要继续研究其他的肿瘤细胞类型是否也存在同样的溶瘤机制。

本研究首次发现了MV-Edm利用其介导的细胞自噬调控NF-κB信号通路从而拮抗细胞凋亡，并将NF-κB信号通路抑制剂PS1145与MV-Edm联合可以协同溶瘤麻疹病毒疫苗株抗肺癌。本研究发现对于不断发展和优化以MV-Edm为基础的溶瘤病毒治疗非常重要，对于其他溶瘤病毒相关溶瘤机制的探讨以及溶瘤手段的不断优化也提供了有力的借鉴。
